# Can securities supervision reduce corporate tax avoidance?

**DOI:** 10.1371/journal.pone.0270883

**Published:** 2022-07-21

**Authors:** Yewei Wu, Bofu Zhang

**Affiliations:** Accounting Dept/PhD in Business Administration/School of Economics and Management, Tongji University, Shanghai, China; National Institute of Public Finance and Policy, INDIA

## Abstract

Based on the special stock exchange comment letter system in China, this paper explores the relationship between the exchange tax-related comment letters and corporate tax avoidance behavior from the standpoint of securities regulation. We document that firms that engage in more aggressive tax avoidance are more likely to receive a tax-related exchange comment letter. Also, relative to firms receiving a non-tax-related comment letter, firms receiving a tax-related comment letter reduce their tax avoidance behaviors in subsequent years. Further study shows that the more the number of questions and the greater the level of detail in the comment letter, the stronger the effect of tax-related comment letters on corporate tax avoidance. After examining the sample with different degrees of political connection, we find that tax-related comment letters inhibit tax avoidance among state-owned enterprises and private enterprises with close political connections. Finally, the monitoring effect of comment letters on corporate tax avoidance is more pronounced in regions where tax administration is weak, suggesting that the comment letter system can be used as a complementary mechanism for tax administration.

## Introduction

Information disclosure of listed companies is an important factor for investors’ decision-making. The standardized information disclosure is the foundation for effective operation and sustainable development in the capital market. False disclosure, misleading statements, or major misrepresentation not only cause losses to investors, but also seriously disrupt the market order, destroy fair competition, and hinder the healthy and orderly development of the capital market [1]. In this regard, it is necessary to establish the information disclosure supervision system of listed companies and to improve the capital market operation mechanism. The exchange comment letter system is an important supervision system in China’s securities market. The exchange issues a letter of inquiry to the listed company for financial reports, restructuring matters, related-party transactions, abnormal stock fluctuations, and media reports, requiring the listed firms to reply to written letters and publicly disclose the reply letter within the prescribed time. For some issues that have not been solved or are not clearly addressed, the exchange would inquire again, aiming to standardize the information disclosure behavior of listed companies and other information disclosure agents, strengthen the management of information disclosure affairs, and protect the legitimate rights and interests of investors.

**Fig 1:**
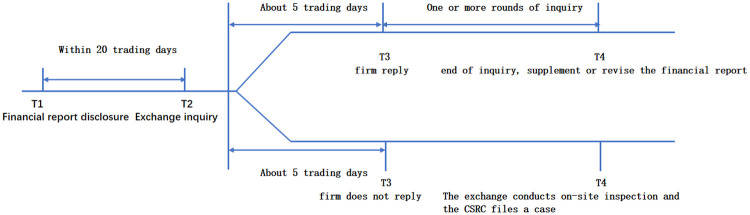
Inquiry process of financial reporting in China’s stock exchanges.

The general process of the Chinese exchange comment letter system is shown in Fig 1. After the listed firms submit their financial report within the exchange system, the exchange would inquire about the firms with irregular financial report disclosure (or insufficient disclosure) within 20 working days, and make the inquiry contents publicly available to the market. The listed firms usually need to respond to the inquiries of the exchange within five working days and disclose the contents to the public at the same time. During one or more rounds of exchanging comment and reply letters, the firm is required to make supplementary disclosure or revision of the financial report errors. If the listed firm fails to reply to the exchange inquiry before the deadline, the exchange would conduct an on-site investigation and might accompany the investigation by China Securities Regulatory Commission (CSRC).

There are several differences between the comment letters system in China and the United States: (1) The regulatory subjects of issuing the comment letters are different. The U.S. Securities and Exchange Commission (SEC) is responsible for issuing comment letters in the U.S., whereas the Chinese stock exchange is responsible for sending the comment letters in China. Since July 2013, China began to implement the information disclosure through an express system, which stipulates for listed firms to register and upload their information disclosure documents through the exchange’s electronic information disclosure system. Thus, the listed firms’ financial information is timely transmitted to the stock exchange, and the exchange is the first regulatory institution to obtain the listed firms’ financial report information. (2) China’s comment letter process is faster. After the completion of the listed firms’ financial reviews, SEC publishes the comment and response letters [2], which is relatively slow. This also resulted in an insignificant U.S. stock market reaction against SEC inquiries [3]. On the contrary, the timely comment letters and instant information disclosure in public can cause significant Chinese stock market reactions [4]. (3) China’s inquiry and regulatory measures are stricter. A considerable proportion of the annual inquiries of China’s stock exchange require third-party organizations to check and issue professional opinions. If listed firms do not comply with the supervision, these firms might be accompanied by on-site investigations as well as China Securities Regulatory Commission filings among any other follow-up supervision means. On the contrary, the SEC inquiry does not require a third-party organization and no follow-up supervision or punishment measures [5]. (4) China’s comment letter system is more decentralized. It issues comment letters by multiple stock exchanges for monitoring different firms, while the comment letter in the US is only issued by the SEC. Through the comparison with the US comment letter system, it can be found that China’s comment letter system should be a more effective ex-post supervision mechanism, and can better regulate the information disclosure behavior of listed companies. Compared with the SEC comment letters system, China’s stock exchange comment letters system demonstrates the characteristics of strong timeliness and wide coverage.

The study by [6] shows that SEC opinion letters can inhibit corporate tax avoidance behaviors. Although in terms of institutional design, the Chinese stock exchange comment letters are timelier and more transparent than those in the United States. However, as a developing economy, China has a poor legal system compared to developed countries. The collusion and rent-seeking behaviors between regulators and listed firms are more common. As a result, the exchange comment letter system may not be able to achieve the expected goals and may be an “ineffective inquiry” made by the Chinese stock exchange to fulfill the tasks assigned by the higher authorities. Therefore, it is necessary to examine the exchange comment letter system in China. From this perspective, our study provides empirical evidence on the relationship between securities regulation and corporate tax avoidance in developing economies. At the same time, there are a large number of state-owned enterprises (SOEs) among Chinese listed firms. SOEs have close ties with government administrators, and the effective stakeholders behind some of these central SOEs are at an even higher administrative rank than the head of the exchange. Chinese Stock exchanges may relax their regulation on SOEs’ tax avoidance practices in consideration of government administrative pressure. In addition, some of the executives of private listed firms used to work in the government, and the political connections established may also affect the effectiveness of the stock exchange in regulating the tax avoidance behavior of listed firms. The Chinese system is fundamentally different from the SEC and is the marginal contribution of this paper. Therefore, under the Chinese institutional environment, whether exchange comment letters can serve as a disincentive to corporate tax avoidance is yet to be tested empirically.

Although the comment letter system has been established for a long time, the Chinese Exchange began to frequently send comment letters to listed companies just in recent years, resulting in the collections and replying observation samples mainly concentrated after the year 2014, which is the main reason for the lack of enough attention. As an important securities market supervision mechanism, it is necessary to conduct a more profound and comprehensive evaluation of the execution effect. The research on the exchange comment letters mainly focuses on the market response, impact on earnings management, stock price crash risk, and merger and acquisition performance [4, 7–9], affirming the positive role of the comment letters on securities supervision. Corporate tax avoidance is a common phenomenon, which causes the loss of tax sources and tax base erosion, especially in the declining growth rate of economic and tax revenue. Under the background of the rigid growth in fiscal expenditure, cracking down on corporate tax avoidance is crucial to guarantee government financial resources and give effective play to various governance functions. After [10] incorporated tax avoidance into the agency problem framework, the tax avoidance behavior is generally regarded as a type I agency problem. Therefore, corporate tax avoidance behavior is not only a challenge in the fiscal and tax fields but also an urgent problem for firms’ financial statements. The exchange comment letter plays a positive role in alleviating the agency problem and improving the quality of accounting information, so can it also play a monitoring role in corporate tax avoidance behavior? Due to the limited resources and capacity of the tax bureau, if the comment letter can play a role in reducing the degree of corporate tax avoidance, the securities supervision can be seen as an important supplement to tax collection and management, which is of great enlightenment to the reform of tax monitoring system. At present, there are few pieces of literature to discuss the relationship between the two, which is related to important issues such as ensuring government financial resources and standardizing the information disclosure behavior of listed companies.

This paper uses theoretical and empirical analysis to test the impact of Shanghai and Shenzhen Stock Exchange tax-related comment letters on corporate tax avoidance. The primary reason for selecting Chinese listed firms as the research object is that extant literature mainly focuses on SEC comment letters [5, 11–13], with a lack of attention on the Chinese stock exchange comment letters. The effectiveness and coverage of the Chinese stock exchange inquiry system may have different regulatory backgrounds compared with the SEC; On the other hand, the monitoring of corporate tax avoidance behavior is an urgent practical problem to be solved by central governments around the world. As the largest developing economy and the second-largest economy in the world, the impact of securities supervision on corporate tax avoidance in the context of China can be significant for the establishment and reform of the international corporate tax monitoring system. The empirical results show firms that engage in greater tax avoidance are more likely to receive a tax-related exchange comment letter, and the firms receiving the tax-related comment letters would reduce tax avoidance behavior in the subsequent fiscal years. Furthermore, we find that the numbers of tax-related comment letter questions have a negative relation to tax avoidance behavior. After considering the impact of government behavior, the tax avoidance effect is found to be more significant for state-owned enterprises (SOEs) and non-SOEs that have political connections, and there exists an alternative relationship for tax avoidance monitoring between tax collection and tax-related comment letters. The above results remained consistent after multiple robustness tests.

The contributions of this paper are: (1) This paper expands the economic consequence evaluation of the exchange comment letters. The previous literature has paid attention to the impact of the comment letter on corporate earnings management, stock price crash risk, and merger and acquisition performance. This paper provides empirical evidence on the relationship between the exchange comment letters and tax avoidance monitoring, which has a positive effect on the comprehensive understanding and evaluation of the exchange comment letter system. (2) This paper has implications for the reform of the tax monitoring system. Under the background of declining economic growth rate and tax revenue, this paper finds a way to monitor tax avoidance to safeguard fiscal resources and maintain the market order. This paper documents that the exchange inquiry system can effectively inhibit the tax avoidance level, and can serve as a useful supplement to the tax collection and management. Therefore, the subsequent reform of the fiscal and tax system should pay close attention to the role of securities supervision in inhibiting tax avoidance, which has a certain reference value for the government and tax authorities to implement the tax collection and management system. (3) This paper enriches the relevant literature on corporate tax avoidance management. Some literature has discussed the factors affecting corporate tax avoidance from government behavior and corporate governance structure perspective, but very few from the perspective of the corporate tax avoidance behavior. This paper provides empirical evidence on the tax avoidance monitoring effect of the exchange comment letter under the securities supervision system, which is an important supplement to the tax avoidance-related literature. (4) Unlike the [6] study, we further discussed the impact of the different types of response letters. Moreover, this paper considers the impact of the government behavior on the tax avoidance monitoring effect of the tax-related comment letter, which is an extension of [6].

## Literature review and hypotheses

### Literature review

The traditional view of corporate tax avoidance as a tool that can increase enterprise value by saving cash flow, while [10] put forward the concept of tax avoidance agency, which believes that the reason why enterprises evade tax is due to agency problems and can reduce the value of the company. This view is widely used in the follow-up research of corporate tax avoidance. By combining the existing literature on corporate tax avoidance, we can classify the factors affecting corporate tax avoidance behavior into internal factors and external factors. Most of the literature also analyzes tax avoidance behavior from the perspective of agency problems.

There is a wide range of internal factors affecting corporate tax avoidance, including CEO salary incentives [14], financial statement transparency [15], insider trading profits [16] and internal control defects [17]. These factors reflect the agency problems of enterprises to a certain extent and can have a significant impact on corporate tax avoidance. The external factors affecting corporate tax avoidance mainly include industry competitive pressure [18], customer-supplier relationship [19], and customer concentration [20].

The extant comment letter literature mainly investigates the effectiveness of the securities supervision system with the nature of the SEC comment letter from the aspects of the information environment, audit pricing, internal control, and rotation of the chief financial officer [11–13]. The common perception is that SEC inquiries can improve corporate governance by reducing the level of information asymmetry. For example, corporate information transparency is improved after receiving SEC comment letters [5]. Also, the information from the comment letter can be fully utilized by analysts to reduce predictive divergence [21], which promoted relevant research in the field of securities supervision and are important for the development of follow-up research. However, only limited literature pays attention to the effect of Chinese stock exchange comment letter regulation on corporate tax avoidance. Tax avoidance is fundamentally different from earning management, internal control quality, and audit pricing. Therefore, the relationship between comment letter supervision and corporate tax avoidance deserves further theoretical and empirical analysis.

### Hypothesis

The exchange comment letter can monitor the tax avoidance behavior of listed companies through the controlling effect and the reputation effect. On the one hand, the exchange continues to pay attention to the information disclosure behavior of listed companies, not only to the standardization and timeliness of information disclosure but also to supervise the specific matters disclosed, including related-party transactions, mergers and acquisitions, performance commitment, goodwill impairment, etc. Corporate tax avoidance is often carried out through complex and fuzzy transactions, which is the key transaction behavior that the exchanges are focused on. The comment letter identifies the problem with the company’s accounting policy choice and the transparency of accounting policy implementation and disclosure, including those key factors related to financial accounting [22]. The regulatory function of the inquiry system can urge listed companies to explain relevant problems in the form of replies, improve the adequacy and detail of information disclosure, reduce the degree of information asymmetry, effectively inhibit the moral risk of management, and help alleviate the type I agency problems [23], and subsequently reduce corporate tax avoidance [10]. In particular, when listed firms involve transfer pricing, thin capitalization, and other tax avoidance behaviors, such firms will be issued comment letters to further explain tax-related matters and may require their rectification and even limit tax avoidance related-party and fuzzy transactions. This reduces the degree of tax avoidance, information asymmetry, and agency cost in the capital market.

On the other hand, the listed company received a comment letter and sent an abnormal signal to the market, which not only damaged corporate value [4] but also attracted more attention from stakeholders, thus forming a stricter external regulatory environment. This spillover effect of positive externalities can drive the expansion of negative information [24], with a bad impact on corporate reputation. In particular, when asked about tax-related issues, it conveys negative signals such as poor quality of corporate accounting information, weak tax compliance, and bad credit status to the market, which may even cause the attention of the tax bureau, and may harm the reputation and operation of the firm. Therefore, after receiving the comment letter, firms have the incentive to reduce the tax avoidance degree and improve information transparency in order to reduce the negative impact of the comment letter.

Based on the above analysis, we hypothesize that the tax-related comment letter can reduce the firm’s tax avoidance behavior.

## Sample and research design

### Sample description

Although the comment letter system was established earlier, the exchange has only sent comment letters frequently since 2014. Therefore, we selected A-share listed companies from 2014 to 2019 as study samples and eliminated (1) financial companies; (2) listed companies with transaction status of special treatment and particular transfer; (3) observation samples with income tax expenses less than 0 and missing relevant financial data. A total of 2489 valid observations were eventually obtained, among which 885 observations received the exchange comment letters and 236 observations received tax-related comment letters. The specific sample distribution is shown in Table 1. It can be seen that the proportion of listed companies receiving tax-related comment letters is increasing over time, with the highest value reached in 2018 at 32.32%. This shows that the stock exchange has strengthened the supervision of information disclosure for listed companies. The relevant data of the comment letters are collected manually through the website of the Shanghai and Shenzhen exchange. The financial data are from the China stock market & Accounting Research Database, and the tax inspection data are from the China financial statistics yearbook.

**Table 1:** Annual sample distribution of comment letters.

Year	Tax-related	Non tax-related	Proportion
2015	25	66	27.47%
2016	37	138	21.14%
2017	42	94	30.88%
2018	64	134	32.32%
2019	68	217	23.86%
Total	236	649	26.67%

### Modelling

Before testing the impact of tax-related comment letters on corporate tax avoidance behaviors, we need to investigate the influencing factors for enterprises receiving tax-related comment letters. For this purpose, we refer to the practice of [6] and construct the Eq (1):

$$\begin{array}{c} Tax\underline{\;\;}inquiry_{i,t}=\alpha_{0}+\alpha_{1}BTD_{i,t}/TS_{i,t}+X_{i,t}+\lambda_{i}+\tau_{i}+\varepsilon_{i,t} \end{array}$$ (1)

Where the subscript *i* represents the firm and *t* represents the year. The dependent variable *Tax*_*inquiry*_*i*,*t*_ equals 1 means the receipt of tax-related comment letters, and 0 is the receipt of non-tax-related comment letters. Specifically, we divide the content of the comment letters into tax-related and non-tax-related letters. When a comment letter contains the keywords “*tax*”, “*invoice*”, “*profit transfer*” and “*thin capitalization*”, then this comment letter is categorized as a tax-related comment letter. Otherwise, it is classified as a non-tax-related comment letter. Furthermore, we also manually check each letter to ensure that the classification is correct. BTD is defined as book-tax differences, which measure the firm’s tax avoidance. We follow [25], which takes BTD = (pre-tax income—(income tax expense-defer tax expense)/taxrate)/lagged total asset, where *taxrate* is not constant at 25%, Some enterprises enjoy preferential tax rates, such as 15%, 10%. Different from the definition of BTD in Kubick et al. (2016), since minority interest is an after-tax account in income report in China accounting standard, so we don’t minus minority interest when calculating BTD. There is a positive relationship between BTD and firms’ tax avoidance behaviors. But the key variable BTD contains earnings management, which would be noise to our analysis. So, we further take TS as another tax avoidance measure. The measure of TS also follows the method of [25, 26], as shown in model (2) and model (3). There is also a positive association between TS and firms’ tax avoidance behaviors. *X*_*i*,*t*_ contains a list of control variables, which contains firms’ finance, operation, and governance structure variables. λ_*i*_, *τ*_*i*_ and *ε*_*i*,*t*_ are industry fixed effect, year fixed effect, and the error term respectively. *Logit* is used to estimate the Eq (1), and the standard errors are clustered at the firm level.

Note that there are still some other methods to measure firms’ tax avoidance such as the effective tax rate method. The reason why we choose BTD and TS as measures of tax avoidance is as follows: The effective tax rate is the tax expenditure of an enterprise divided by the pre-tax income. The more tax evasion, the lower the effective tax rate. This method is widely used in the literature related to tax evasion [27]. However, the effective tax rate method has obvious disadvantages. When enterprises evade taxes by manipulating pre-tax income and pre-tax profit at the same time, the tax expenditure and pre-tax profit of enterprises will change in proportion. At this time, the effective tax rate can not effectively reflect the tax avoidance behavior of enterprises. Based on this, the literature has gradually turned to the book taxable income method to measure the tax avoidance of enterprises. Different from the effective tax rate method, the book-tax differences method mainly depends on the systematic decomposition of accounting book income and taxable income. The logic behind it is that business operators have incentives to report more real income to shareholders, and they also have incentives to hide income from tax authorities. The former is generally the book income of the enterprise, while the latter is taxable income, and the degree of concealing income from tax authorities will be reflected in the difference between the two. Therefore, in addition to the differences in normal tax accounting systems, the differences between book income and taxable income reflect the degree of tax avoidance of enterprises to a certain extent [25].

$$\begin{array}{c} BTD_{i,t}=\alpha_{0}+\alpha_{1}TA_{i,t}+\mu_{i}+\varepsilon_{i,t} \end{array}$$ (2)

$$\begin{array}{c} TS=\mu_{i}+\varepsilon_{i,t} \end{array}$$ (3)

Where *TA*_*i*,*t*_ is total accruals for firm *i* in year *t*, scaled by the lagged value of assets. TS is the residual of Eq (2). For Eq (2), we use the annual firm fix effect estimation by industry and year. The variable TS can exclude the impact of earnings management to some extent, hence offering a clear analysis to our test.

In order to test the hypothesis, we construct the Eq (4) for testing:

$$\begin{array}{c} BTD_{i,t}/TS_{i,t}=\beta_{0}+\beta_{1}taxfirm*post+X_{i,t}+\mu_{i}+\lambda_{i}+\tau_{i}+\varepsilon_{i,t} \end{array}$$ (4)

The dependent variables in Eq (4) are the same as the independent variables of Eq (1). *taxfirm* is a dummy variable that equals 1 if a firm received a tax-related comment letter at any point during our sample. post is a dummy variable equaling 1 for fiscal years after receiving a tax-related comment letter. *X*_*i*,*t*_ contains control variables same as model (1). *μ*_*i*_ represent firm fix effect, λ_*i*_, *τ*_*i*_ and *ε*_*i*,*t*_ are industry fixed effect(Some listed firms changed their industries within the sample interval, and the use of firm fixed effects did not complete the absorption of industry fixed effects, so this paper controls for industry fixed effects.), year fixed effect, and the error term respectively. Model (4) is a stagger Difference-in-Differences model. post will be omitted since complete collinearity with taxfirm*post. *taxfirm* would be omitted for firm fix effect, so there is only an interactive term remaining in the equation. We take the standard errors clustered at the firm level when estimating the model and winsorizing 1% of all continuous variables to avoid extreme value interference. Specific variables are defined in Table 2.

**Table 2:** Variables definitions.

Variable	Definition
BTD	(Pretax income–(income tax expense-defer tax expense)/tax rate)/lagged total asset
TS	See Eqs (2) and (3)
ETR	Income tax expense/pretax income
CETR	Cash taxes paid/pretax income
Tax_inquiry	Equal 1 when firm receiving tax-related comment letter, and 0 otherwise.
taxfirm	Equal 1 if a firm received a tax-related comment letter at any point during our sample, otherwise 0.
post	Equal 1 for fiscal years after receiving tax-related comment letter, otherwise 0
Enfojjk rce	Tax inspection income / tax income (province level)
political connection	Equal 1 if the shareholders or executives served in a government agency, otherwise 0
Inquiry_question	Total number of questions in the comment letter received that year
Inquiry_detail	Number of comment letter sentences / Number of comment letter questions
revise	Equal 1 for revise financial report information, supplementary or explanatory information equals 0
size	Natural logarithm of total assets
lev	Total liabilities / total assets
roi	Investment income /Year-end total assets
mb	Market value / Book value
ppe	Net fixed assets / Total assets
intang	Net intangible assets / total assets
invent	Net inventory / Total assets
age	The natural logarithm of the year of establishment plus 1
soe	1 if it is a state-owned enterprise, otherwise 0
equity_concen	The largest shareholder shareholding ratio
boardsize	The natural logarithm of board members
iboardratio	Number of independent directors / board size

Table 3 presents the descriptive statistics of the main variables. The average of book-tax differences (BTD) is negative. It indicates that the overall earnings pretax profit is less than the taxable income. And the average TS is also negative. The average value of *Tax_inquiry* is 0.267, which means about 26.7% of firms has received tax-related comment letter. Within the inquiry sample, the average number of inquiry questions (*Inquiry_question*) is 8.89. For each question, the firm replies to nearly 17 sentences per question. The average round of inquiry is 1.1 (*Inquiry_num*). In addition, about 21.3% of the enterprises in the sample were state-owned enterprises (*soe*), with the average largest shareholder shareholding ratio (*Equity_concen*) of 29.5%, and the number of independent directors representing over a third of board members (*Iboardratio*).

**Table 3:** Descriptive statistics of the main variables.

variable	N	mean	sd	p25	p50	p75
BTD	2,489	-0.002	0.009	-0.003	-0.001	0.001
TS	2,489	-0.012	0.072	-0.036	-0.002	0.021
ETR	2,489	0.167	0.183	0.065	0.153	0.245
CETR	2,489	0.635	0.761	0.226	0.512	1.005
Tax_inquiry	885	0.267	0.442	0.000	0.000	1.000
verify	885	0.376	0.485	0.000	0.000	1.000
Inquiry_question	885	8.965	4.021	6.000	9.000	11.000
Inquiry_num	885	1.108	0.389	1.000	1.000	1.000
revise	885	0.376	0.485	0.000	0.000	1.000
size	2,489	22.158	1.188	21.334	22.086	22.898
roi	2,489	0.008	0.017	0.000	0.002	0.008
mb	2,489	2.570	2.545	0.915	1.712	3.261
lev	2,489	0.449	0.215	0.276	0.440	0.610
ppe	2,489	0.182	0.156	0.052	0.144	0.270
invent	2,489	0.135	0.137	0.042	0.098	0.174
intang	2,489	0.046	0.052	0.015	0.032	0.056
age	2,489	2.979	0.264	2.833	2.996	3.178
soe	2,489	0.213	0.410	0.000	0.000	0.000
Equity_concen	2,489	0.295	0.131	0.193	0.278	0.375
boardsize	2,489	2.086	0.203	1.946	2.197	2.197
Iboardratio_ratio	2,489	0.382	0.056	0.333	0.364	0.429
enforce	2,435	0.025	0.021	0.013	0.022	0.030

## Results

### Baseline regressions

Table 4 shows the regression results of Eq (1). The coefficient of BTD in Column (1) is positive at the 1% level, which means firms that engage in more aggressive tax avoidance would be more likely to receive tax-related comment letters. The coefficient of TS in Column (2) is positive but at the 10% level, which also suggests firms that engage in more aggressive tax avoidance would be more likely to receive tax-related comment letters.

**Table 4:** Tax avoidance and likelihood of receiving tax-related comment letter.

	(1)	(2)
	Tax_inquiry	
BTD	3.114***	
	(2.886)	
TS		2.401*
		(1.846)
size	0.056	0.072
	(0.546)	(0.697)
roi	-1.651	-0.668
	(-0.357)	(-0.145)
mb	0.016	0.017
	(0.328)	(0.340)
lev	0.736	0.519
	(1.636)	(1.171)
ppe	0.758	0.485
	(1.101)	(0.709)
invent	-0.487	-0.354
	(-0.593)	(-0.431)
intang	-0.013	-0.265
	(-0.008)	(-0.156)
age	0.092	0.125
	(0.252)	(0.343)
soe	0.057	0.073
	(0.245)	(0.315)
Equity_concen	0.637	0.685
	(0.914)	(0.987)
boardsize	0.349	0.367
	(0.653)	(0.689)
iboardratio	-0.667	-0.604
	(-0.360)	(-0.327)
year	yes	yes
ind	yes	yes
Constant	-17.248	-17.639
	(-0.036)	(-0.037)
N	850	850
Pseudo R2	0.075	0.069

Note:

*, **, *** indicate the significance levels of 10%, 5%, and 1%, respectively, and the z-values in parentheses adjusted for firm-level clustering. The regression sample is less than the description because there exist all firms in one industry that does not receive any tax-related comment letter, and the logit model will automatically exclude those samples.

Table 5 shows the regression results of Eq (4). The coefficient of taxfirm*post in Column (1) is negative at the 1% significant level, which means that compared with firms receiving non-tax-related comment letters, tax-related comment letters can reduce the tax avoidance behavior of listed firms in subsequent years. While excluding the influence of earning management, the coefficient of taxfirm*post in Column (2) is negative at the 1% significant level, which also suggests tax-related comment letter exerts the role of reducing tax avoidance.

**Table 5:** The effect of tax-related comment letters on subsequent tax avoidance.

	(1)	(2)
	BTD	TS
taxfirm*post	-0.022***	-0.028***
	(-3.156)	(-4.682)
size	0.039***	0.021***
	(6.992)	(4.524)
roi	0.880***	0.559***
	(7.576)	(5.675)
mb	0.002	0.002
	(1.266)	(1.348)
lev	-0.232***	-0.131***
	(-13.321)	(-8.885)
ppe	-0.090***	-0.022
	(-3.189)	(-0.936)
invent	0.013	-0.013
	(0.401)	(-0.461)
intang	-0.027	0.051
	(-0.370)	(0.828)
age	-0.070	-0.038
	(-0.951)	(-0.603)
soe	-0.003	-0.002
	(-0.227)	(-0.159)
Equity_concen	0.072*	0.059*
	(1.904)	(1.844)
boardsize	0.052**	0.056***
	(2.546)	(3.193)
iboardratio	0.140**	0.099*
	(2.134)	(1.780)
firm	yes	yes
year	yes	yes
ind	yes	yes
Constant	-0.640**	-0.458**
	(-2.404)	(-2.029)
N	2,489	2,489
Adj R2	0.339	0.334

Note:

*, **, *** indicate the significance levels of 10%, 5%, and 1%, respectively, and the t-values in parentheses adjusted for firm-level clustering.

The premise of using difference-in-differences is to satisfy parallel trends, for which we constructed the following model to verify:

$$\begin{array}{c} \begin{array}{c} BTD_{i,t}/TS_{i,t}=\gamma_{0}+\sum_{i=-3}^{-1}\gamma_{i}pre_{i}+current*taxfirm+\\ \sum_{j=1}^{4}\gamma_{j}post_{j}+X_{i,t}+\mu_{i}+\lambda_{i}+\tau_{i}+\varepsilon_{i,t} \end{array} \end{array}$$ (5)

In the Eq (5), *pre*_*i*_ is the interaction term between *taxfirm* and dummy variable that equals 1 for the *i* year before the tax-related comment letter was received, and 0 otherwise. *current* takes 1 for the year the tax-related comment letter is received, and 0 otherwise. *post*_*j*_ is the interaction term between *taxfirm* and dummy variable that equals 1 for *j* years after the comment letter is received, and 0 otherwise. Based on the 4 years before the receipt of the tax-related inquiry letter. The regression results are shown in Table 6. The coefficients of all *pre*_*i*_ are not significant. It implies that there is no significant difference in terms of tax avoidance degree between the two types of enterprises before receiving the tax-related comment letters, so the parallel trend assumption is satisfied. The coefficient *current* in column (2) is significantly negative, indicating that the tax-related comment letter plays a key role in reducing tax avoidance. The coefficients of all *post*_*j*_ in columns (1) and (2) are significantly negative, indicating that tax-related comment letters can continue to reduce corporate tax avoidance.

**Table 6:** Dynamic effect.

	(1)	(2)
	BTD	TS
pre_3	-0.011	-0.013
	(-0.923)	(-1.331)
pre_2	-0.016	-0.020
	(-0.809)	(-1.241)
pre_1	0.013	0.010
	(1.431)	(1.329)
current	-0.012	-0.021***
	(-1.273)	(-2.616)
post_1	-0.029**	-0.034***
	(-2.531)	(-3.461)
post_2	-0.029**	-0.026**
	(-2.075)	(-2.185)
post_3	-0.035**	-0.031**
	(-2.002)	(-2.090)
post_4	-0.056*	-0.061**
	(-1.797)	(-2.303)
controls	yes	yes
firm	yes	yes
year	yes	yes
ind	yes	yes
Constant	-0.604**	-0.440*
	(-2.262)	(-1.944)
N	2,489	2,489
Adj R2	0.341	0.336

Note:

*, **, *** indicate the significance levels of 10%, 5%, and 1%, respectively, and the t-values in parentheses adjusted for firm-level clustering.

### Robustness tests

#### Propensity-score matching and Difference-in-Differences

Estimation bias may occur due to systematic differences between firms that received tax-related and non-tax-related comment letters. Thus, the propensity-score matching (PSM) method is used to control the differences between these two categories of firms. Specifically, we used the 1:1 nearest neighbor pairing method with replacement and a caliper distance set to 0.1 to select the closest control sample of the firms that received the tax-related comment letter from those that received non-tax-related comment letters. Finally, 593 observations with tax-related comment letters and 575 observations with non-tax-related comment letters were obtained. Table 7 shows the mean difference test of firms’ characteristic-related variables between propensity-score matching sample treatment group and the control group. There is no significant difference in covariates between the treatment group (firms that receive tax-related comment letters) and the control group (firms that receive non-tax-related comment letters).

**Table 7:** Covariate balance of PSM sample.

	Control		Treat		
	N	Mean	N	Mean	MeanDiff
size	575	22.244	593	22.172	0.072
roi	575	0.009	593	0.009	0.000
mb	575	2.551	593	2.547	0.004
lev	575	0.478	593	0.469	0.009
ppe	575	0.182	593	0.189	-0.007
invent	575	0.133	593	0.137	-0.005
intang	575	0.046	593	0.046	0.000
age	575	2.995	593	2.988	0.007
soe	575	0.245	593	0.253	-0.008
Equity_concen	575	0.300	593	0.299	0.001
boardsize	575	2.096	593	2.098	-0.003
iboardratio	575	0.382	593	0.381	0.001

**Fig 2:**
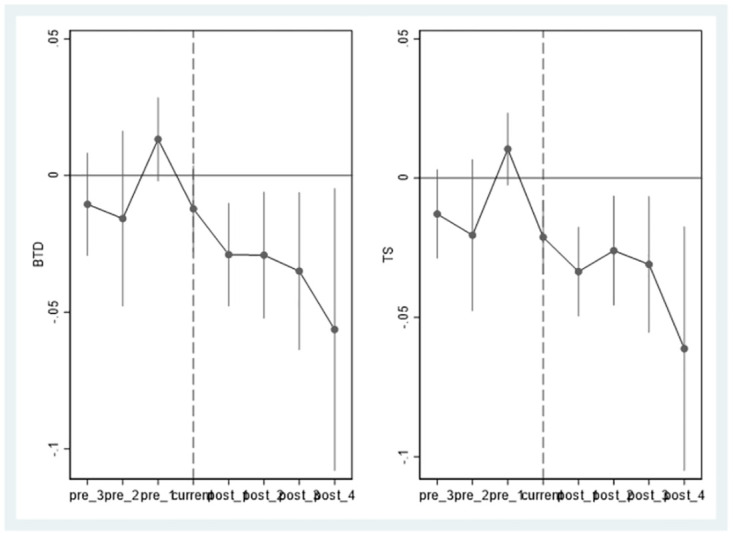
Dynamic effect of stagger DID.

**Fig 3:**
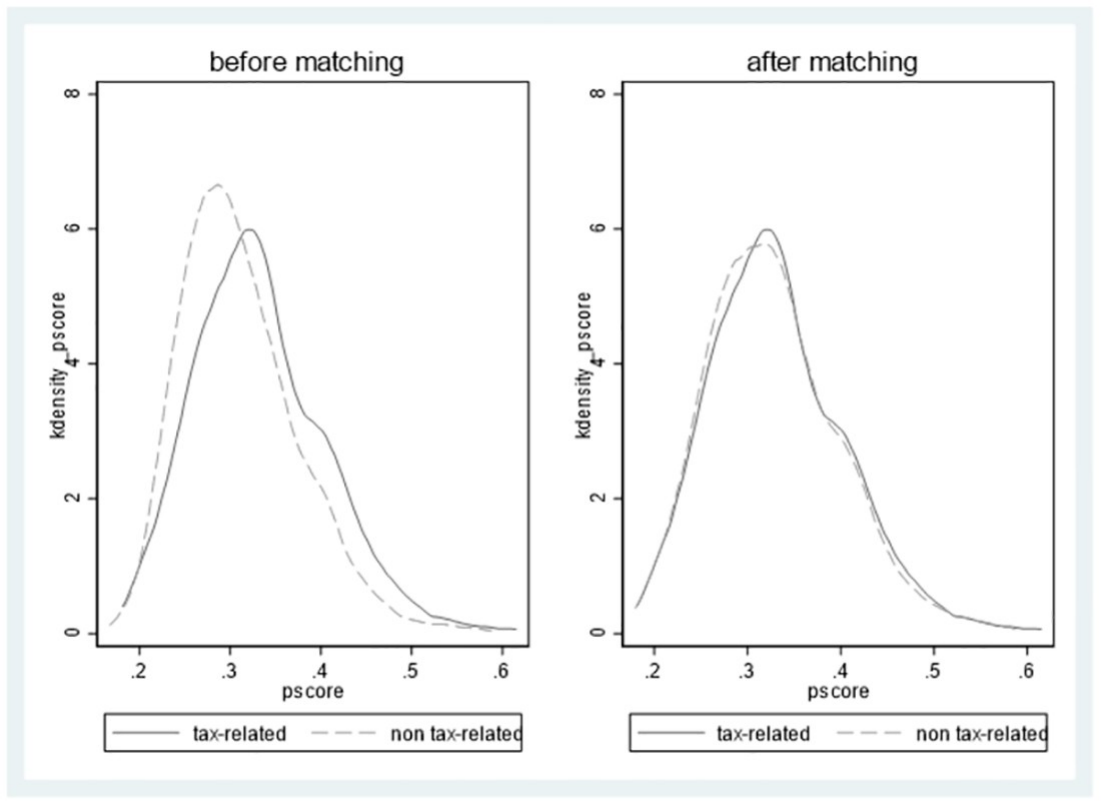
Kdensity pscore of sample before and after PSM.

**Fig 4:**
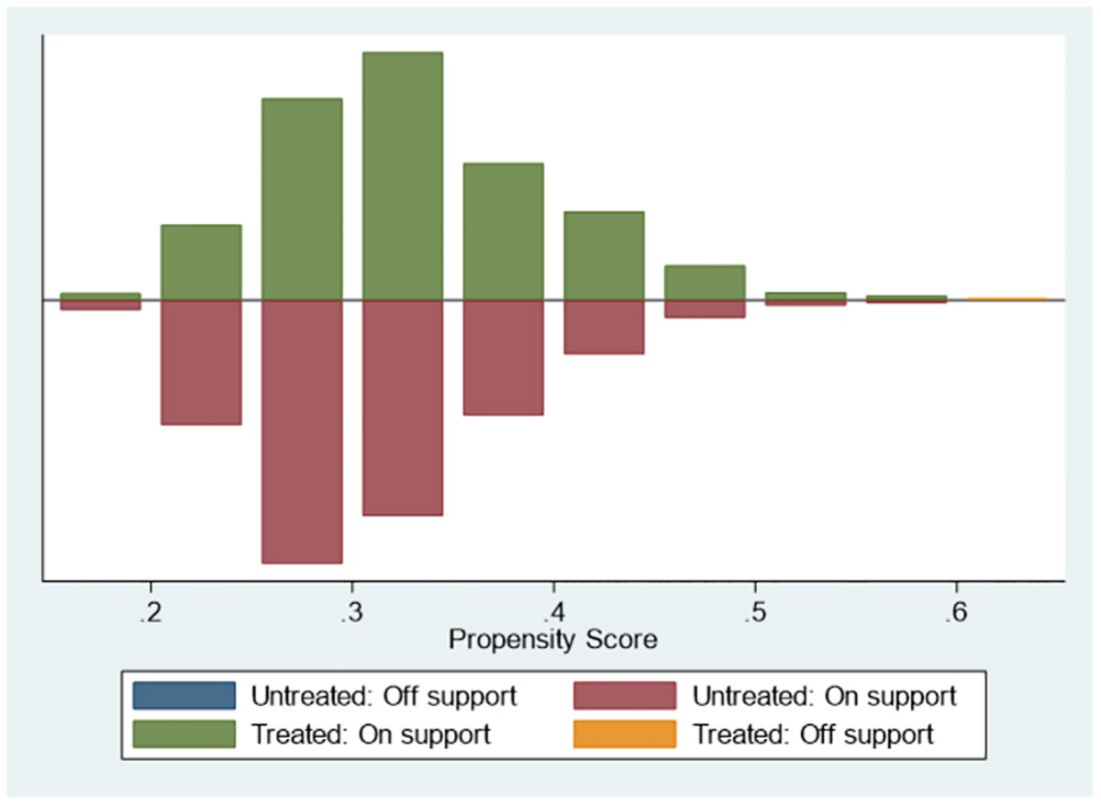
Matching result diagram. The regression results based on PSM samples are shown in Table 8. The coefficient of taxfirm*post is significantly negative in both Column(1) and Column(2), indicating that the conclusions of baseline still hold after controlling the difference of treated and controlled groups.

The key variable of this paper explains whether the firm receives an inquiry (*inquiry*) is a dummy variable, and the two types of enterprises may have varied greatly, leading to the self-selection problem. This paper applies PSM to two classes of enterprises for all control variables in the benchmark regression. We used one-to-one nearest no-back pairing with a caliper set to 0.01. Eventually, we obtain 869 inquiry samples and 869 non-inquiry samples. Fig 2 shows the K-density P score of the sample before and after PSM. After the matching, a tax-related sample and a non-tax-related sample are almost coincident. Figs 3 and 4 show the matching results. We calculate the t-value of all control variables between treatment (*inquiry*) and control (*non-inquiry*) samples. The findings show that there is no significant difference between the main variables of the two types of firms, indicating that the pairing is effective.

The regression results based on PSM samples are shown in Table 8. The coefficient of taxfirm*post is significantly negative in both Column (1) and Column (2), indicating that the conclusions of the baseline still hold after controlling for the difference between the treatment and control groups.

**Table 8:** PSM sample regression results.

	(1)	(2)
	BTD	TS
taxfirm*post	-0.019*	-0.027***
	(-1.907)	(-3.043)
controls	yes	yes
firm	yes	yes
year	yes	yes
ind	yes	yes
Constant	-0.966**	-0.519
	(-2.044)	(-1.276)
N	1,168	1,168
Adj R2	0.317	0.319

Note:

*, **, *** indicate the significance levels of 10%, 5%, and 1%, respectively, and the *t-values* in parentheses adjusted for firm-level clustering.

#### Placebo test

To prevent the interference of unexpected results, we conducted 1000 random number placebo tests. Fgi 5 shows that most t-value of coefficient for the stagger DID interaction term are within the dotted line (suggesting that the coefficient of DID is insignificant), indicating random shock is unlikely to cause the result.

**Fig 5:**
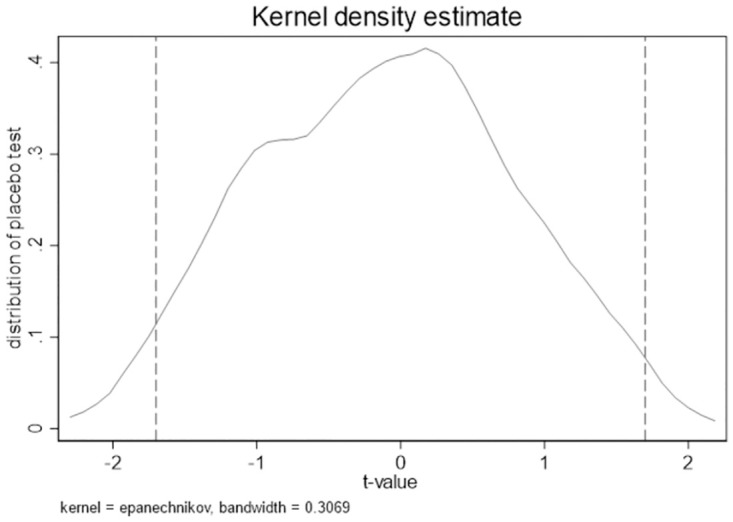
Placebo test.

#### The alternative measures

Although the book-tax differences (BTD) measure has some advantages in measuring corporate tax avoidance, the effective tax rates (ETR) method is still the most popular tax avoidance measurement method. In this regard, we constitute the ETR and Cash ETR(In China, Cash ETR includes value-added tax, which is the number one tax in China, while ETR only includes corporate income tax, so the average value of CETR is much higher than ETR.) in which ETR = income tax expense / pre-tax profit, CETR = tax paid / pre-tax profit, and re-estimate the model(4). The results are shown in Table 9. The coefficient of taxfirm*post is significantly positive in Column (1), which means receiving tax-related comment letters can improve ETR and reduce firms’ tax avoidance behaviors. But the coefficient of taxfirm*post is not significant in Column (2), suggesting receiving tax-related comment letters can not impact cash ETR.

**Table 9:** Effect of tax-related comment letter on ETR and cash ETR.

	(1)	(2)
	ETR	CETR
taxfirm*post	0.028**	0.123
	(2.115)	(1.361)
size	0.015***	-0.062*
	(3.201)	(-1.808)
roi	0.541	0.782
	(1.623)	(0.462)
mb	-0.004	-0.062***
	(-1.575)	(-4.362)
lev	-0.073***	-0.043
	(-3.028)	(-0.251)
ppe	-0.100***	0.056
	(-2.933)	(0.219)
invent	0.078**	0.780**
	(1.991)	(2.513)
intang	-0.013	0.088
	(-0.160)	(0.160)
age	0.039**	0.218*
	(2.546)	(1.801)
soe	0.005	0.238***
	(0.390)	(2.619)
Equity_concen	0.044	0.179
	(1.370)	(0.753)
boardsize	-0.003	-0.199
	(-0.130)	(-0.891)
iboardratio	0.014	0.292
	(0.171)	(0.332)
firm	yes	yes
year	yes	yes
ind	yes	yes
Constant	-0.388***	1.289
	(-2.860)	(1.142)
N	2,489	2,489
Adj R2	0.062	0.041

Note:

*, **, *** indicate the significance levels of 10%, 5%, and 1%, respectively, and the *t-values* in parentheses adjusted for firm-level clustering.

#### Tax policy shocks

Relevant tax policy adjustments during the sample period may interfere with the estimation results. Controlling the interaction of provinces and years (province*year) and the interaction of industry and year (industry*year) can eliminate the interference of industry tax policy and regional tax policy, the regression results are shown in Table 10. The coefficient of taxfirm*post both in Column (1) and Column (2) is still significantly negative, which means that after controlling industry tax policy and regional tax policy, our results are still robust.

**Table 10:** Controls the impact of tax policy impacts.

	(1)	(2)
	BTD	TS
taxfirm*post	-0.025***	-0.030***
	(-3.270)	(-4.698)
size	0.035***	0.022***
	(5.924)	(4.440)
roi	0.812***	0.523***
	(6.645)	(5.038)
mb	0.002	0.003*
	(1.415)	(1.931)
lev	-0.222***	-0.121***
	(-12.156)	(-7.792)
ppe	-0.097***	-0.037
	(-3.193)	(-1.419)
invent	0.015	-0.024
	(0.425)	(-0.793)
intang	-0.009	0.039
	(-0.108)	(0.565)
age	-0.068	-0.019
	(-0.856)	(-0.286)
soe	0.005	0.005
	(0.381)	(0.407)
Equity_concen	0.065	0.049
	(1.613)	(1.435)
boardsize	0.050**	0.045**
	(2.246)	(2.379)
iboardratio	0.137*	0.086
	(1.943)	(1.435)
firm	yes	yes
year	yes	yes
ind	yes	yes
province*year	yes	yes
industry*year	yes	yes
Constant	-0.650**	-0.509**
	(-2.193)	(-2.024)
N	2,489	2,489
Adj R2	0.354	0.347

Note:

*, **, *** indicate the significance levels of 10%, 5%, and 1%, respectively, and the *t-values* in parentheses adjusted for firm-level clustering.

## Further analyses

### Securities supervision strength

There are cases in the sample where firms were asked multiple times in a year and received comment letters for different financial reports (annual and quarterly reports). The multiple inquiries and multiple types of letters of questioning highlight the strength of exchange supervision. Does the stronger intensity lead to less corporate tax avoidance? In this regard, this paper examines the impact of regulatory intensity on corporate tax avoidance behavior by using the sample of inquired firms. We construct two variables the rounds of inquiries (*inquiry_num*) and the number of inquiry questions (*inquiry_question*) as proxy variables for regulatory intensity.

Table 11 shows the regression results of the impact of tax-related comment letter text information on tax avoidance behaviors. The regression results do not contain firms that receive non-tax-related comment letters. The results of Column (1) and Column (3) show that the coefficient *Inquiry_question* is significantly negative, indicating that the more number of questions in the comment letter, the greater effect on reducing tax avoidance; Column (2) shows that the greater level of detail in the comment letter (*Inquiry_detail*), the lower the tax avoidance; However, the coefficient of *Inquiry_detail* in Column (4) is not significant.

**Table 11:** The impact of tax-related comment letter text information.

	(1)	(2)	(3)	(4)
	BTD		TS	
Inquiry_question	-0.121**		-0.021*	
	(-2.430)		(-1.725)	
Inquiry_num		-0.087***		-0.028
		(-2.883)		(-1.005)
controls	yes	yes	yes	yes
firm	yes	yes	yes	yes
year	yes	yes	yes	yes
ind	yes	yes	yes	yes
Constant	17.477*	3.412	-0.227	1.469
	(1.949)	(1.300)	(-1.215)	(0.609)
N	236	236	236	236
Adj R2	0.684	0.635	0.172	0.589

Note:

*, **, *** indicate the significance levels of 10%, 5%, and 1%, respectively, and the t-values in parentheses adjusted for firm-level clustering.

### Reply letters

After receiving the tax-related comment letters, there are two different types of responses to the exchange comment letters. One type of response is to mandatorily revise the financial reporting information, and the other is asked to supplement the additional financial information. The revision request is sent when the firm’s previous financial disclosures contain accounting errors. In this case, the firm needs to adjust its financial reporting behaviors. On the other hand, the supplementary request is sent when the firm needs to provide additional financial information disclosure. In this case, the issue might only be inadequate disclosure. Therefore, we expect that the revision case in the reply letter would better reduce the degree of corporate tax avoidance. The variable revise equals 1 indicating the firms are required to revise the financial reporting information, whereas 0 indicates the firms are required to supplement the explanatory financial reporting information.

Table 12 shows the regression results of the firms’ revised accounting information, and the sample only includes firms that received the tax-related comment letters. The coefficient *revise* in Columns (1) and (2) is significantly negative, indicating that the revision type of the comment letter is more statistically significant in reducing the degree of corporate tax avoidance than the supplementary one.

**Table 12:** Impact of revising accounting information.

	(1)	(2)
	BTD	TS
revise	-0.035**	-0.077**
	(-2.451)	(-3.087)
size	0.016**	-0.267
	(2.372)	(-1.254)
roi	0.514	1.512
	(1.542)	(1.608)
mb	0.005	-0.001
	(1.507)	(-0.182)
lev	-0.158***	-0.076
	(-5.157)	(-0.479)
ppe	-0.050	0.064
	(-1.142)	(0.361)
invent	0.095*	-0.383
	(1.719)	(-1.104)
intang	-0.145	-3.923
	(-1.102)	(-1.326)
age	0.017	-0.298
	(0.644)	(-0.385)
Equity_concen	-0.026	-0.098
	(-0.505)	(-0.203)
boardsize	0.008	0.013
	(0.231)	(0.071)
iboardratio	-0.056	-0.511
	(-0.416)	(-0.809)
firm	yes	yes
year	yes	yes
ind	yes	yes
Constant	-0.380*	7.126
	(-1.804)	(1.087)
N	236	236
Adj R2	0.189	0.764

Note:

*, **, *** indicate the significance levels of 10%, 5%, and 1%, respectively, and the *t-values* in parentheses adjusted for firm-level clustering.

### Political connections

China’s legal system and financial system are weak [28], and the government plays an important role in economic affairs. When the establishment is closely linked to the government, corporate violations are less vulnerable to the relevant punishment [29]. Due to the equity nature, the government provides an “implicit guarantee” for the operation behavior of state-owned enterprises, making less punishment by tax administration for firms that take tax avoidance behaviors. As an institution under the vertical jurisdiction of the China Securities Regulatory Commission, the exchange is less affected by local governments and is highly independent. At the same time, the functionality of the exchange is not to maintain the development of the state-owned enterprises, but to regulate the financial reporting disclosure behavior of the listed companies. Thus, the exchange does not protect the state-owned enterprises. Therefore, with regard to the tax avoidance behavior of state-owned enterprises, the exchange comment letter could play a better monitoring role than the local tax authorities. Under these circumstances, we divided the samples of the model (4) into state-owned enterprises and non-state-owned enterprises based on the ownership, and the results are shown in Table 13.

**Table 13:** Effect of tax-related comment letters on firms’ tax avoidance among different ownership.

	(1)	(2)	(3)	(4)
	BTD		TS	
	SOE	non-SOE	SOE	non-SOE
taxfirm*post	-0.046***	-0.013	-0.061***	-0.019***
	(-3.404)	(-1.506)	(-5.072)	(-2.688)
controls	yes	yes	yes	yes
firm	yes	yes	yes	yes
year	yes	yes	yes	yes
ind	yes	yes	yes	yes
Constant	-1.209**	-0.750**	-0.416	-0.566**
	(-2.260)	(-2.396)	(-0.866)	(-2.168)
N	531	1,958	531	1,958
Adj R2	0.369	0.353	0.398	0.344
P-value	0.060		0.010	

Note:

*, **, *** indicate the significance levels of 10%, 5%, and 1%, respectively, and the *t-values* in parentheses adjusted for firm-level clustering.

The coefficient taxfirm*post in Column (1) is significantly negative. Although the taxfirm*post coefficient in Column (2) is negative, it is not statistically significant. There are significant inter-group differences between these two coefficients (P-value = 0.060), indicating that the role of tax-related comment letters in reducing tax avoidance behaviors mainly exists in state-owned enterprises. The coefficient taxfirm*post of Columns (3) and (4) is significantly negative, but the coefficient in the SOE group is significantly greater than that of the non-state-owned enterprises. There are significant inter-group differences between these two coefficients (*p* − *value* = 0.010), so the result also shows that the exchange comment letter has a better monitoring effect on the tax avoidance behavior of state-owned enterprises.

Non-state-owned enterprises, whose managers or shareholders used to serve in the government, have also formed a political connection with the government. This type of company not only enjoys a more relaxed tax collection and management environment but also reduces the degree of punishment for violations [30]. Compared with the tax authorities, the stock exchanges have better independence, so they can play a better monitoring role on corporate tax avoidance for non-state owned enterprises with political relations. In this regard, we divided non-state-owned enterprises into two groups: political and non-political connection groups. The results are shown in Table 14. For BTD, the coefficient taxfirm*post is only significantly negative in politically connected enterprises. For TS, the absolute value of the coefficient taxfirm*post is higher (*p* − *value* = 0.090). Therefore, among non-state-owned enterprises, tax-related comment letters mainly act on politically connected enterprises.

**Table 14:** The impact of political connections.

	(1)	(2)	(3)	(4)
	BTD		TS	
	Political connection	Non-political connection	Political connection	Non-political connection
taxfirm*post	-0.026*	-0.017	-0.032**	-0.017*
	(-1.694)	(-1.472)	(-2.421)	(-1.890)
size	0.070***	0.040***	0.029**	0.023***
	(4.729)	(4.871)	(2.212)	(3.423)
roi	0.899***	0.825***	0.490*	0.322**
	(2.988)	(5.028)	(1.860)	(2.440)
mb	0.003	0.000	0.004	0.001
	(1.059)	(0.250)	(1.303)	(0.861)
lev	-0.246***	-0.189***	-0.159***	-0.107***
	(-6.170)	(-7.265)	(-4.564)	(-5.117)
ppe	0.030	-0.102**	0.019	-0.001
	(0.428)	(-2.239)	(0.307)	(-0.024)
invent	0.038	0.021	0.038	-0.006
	(0.445)	(0.415)	(0.512)	(-0.151)
intang	-0.320**	0.138	-0.256**	0.179*
	(-2.178)	(1.119)	(-1.983)	(1.807)
age	-0.145	-0.182	-0.150	-0.090
	(-0.847)	(-1.510)	(-1.001)	(-0.925)
Equity_concen	0.030	0.129**	-0.058	0.065
	(0.321)	(2.245)	(-0.719)	(1.412)
boardsize	0.070	0.043	0.099**	0.037
	(1.301)	(1.426)	(2.093)	(1.510)
iboardratio	0.349**	0.095	0.281*	0.048
	(2.011)	(0.981)	(1.846)	(0.622)
firm	yes	yes	yes	yes
year	yes	yes	yes	yes
ind	yes	yes	yes	yes
Constant	-1.260**	-0.398	-0.414	-0.335
	(-2.006)	(-0.954)	(-0.753)	(-0.998)
N	641	1,317	641	1,317
Adj R2	0.295	0.373	0.280	0.387
p-value	0.470		0.090	

Note:

*, **, *** indicate the significance levels of 10%, 5%, and 1%, respectively, and the t-values in parentheses adjusted for firm-level clustering.

### Tax collection and administration

The above results indicate that the tax-related comment letter can be used as an external mechanism to reduce corporate tax avoidance behaviors. Then there might exist a confounding effect between the exchange comment letters and the collection and administration of the tax authorities. [10] show that enhanced tax collection and management (*tax enforcement*) not only reduce shareholders’ wealth but also reduce the encroachment of shareholders’ interests. Thus, the effect of tax-related comment letters can also alleviate agency problems. Therefore, we conjecture that there is a certain substitution relationship between tax-related comment letters and tax enforcement. When the intensity of tax collection and management in the local area is high, the tax avoidance monitoring effect by the exchange comment letter is weak. To this end, we follow [31] to construct the collection and management intensity variable (*enforce*) and divide the sample based on median of *enforce*.

$$\begin{array}{c} T_{p,t}/GDP_{p,t}=\beta_{0}+\beta_{1}IND1_{p,t}/GDP_{p,t}+\beta_{2}IND2_{p,t}/GDP_{p,t}+\beta_{3}OPEN_{p,t}/GDP_{p,t} \end{array}$$ (6)

where the subscript *p* denotes province and *t* denotes year. *t*_*p*,*t*_/*GDP*_*p*,*t*_ is the ratio of tax revenue to GDP; *IND*1_*p*,*t*_/*GDP*_*p*,*t*_ is the ratio of the output value in the primary sector to GDP; *IND*2_*p*,*t*_/*GDP*_*p*,*t*_ is the ratio of the output value in the secondary sector to GDP; *OPEN*_*p*,*t*_/*GDP*_*p*,*t*_ is the ratio of total import and export trade to GDP ratio. The estimated expected tax revenue *T*_*p*,*t*_/*GDP*_*p*,*t*__est is obtained by model (6). Tax collection intensity TE is the ratio of actual tax revenue *T*_*p*,*t*_/*GDP*_*p*,*t*_ to expected tax revenue *T*_*p*,*t*_/*GDP*_*p*,*t*__est.

$$\begin{array}{c} TE_{p,t}=\left(T_{p,t}/GDP_{p,t}\right)/\left(T_{p,t}/GDP_{p,t}\_est\right) \end{array}$$ (7)

The higher the *TE*_*p*,*t*_ ratio, the higher the intensity of tax collection in the region. Table 15 shows the regression results for tax collection and management. The results show that in areas with low tax administration, the exchange comment letter plays a significant role in inhibiting corporate tax avoidance, while in firms with high tax administration intensity, the exchange comment letter does not play a significant role in inhibiting corporate tax avoidance. This indicates a substitution relationship, that is, the exchange comment letter can play a greater role when the tax administration is weak.

**Table 15:** The intensity of tax collection and administration.

	(1)	(2)	(3)	(4)
	BTD		TS	
	*low enforce*	*high enforce*	*low enforce*	*high enforce*
taxfirm*post	-0.030***	-0.013	-0.031***	-0.016
	(-3.163)	(-0.775)	(-3.791)	(-0.889)
Size	0.058***	0.044***	0.026***	0.016
	(6.327)	(2.715)	(3.189)	(1.207)
Roi	1.052***	0.960***	0.494***	0.700**
	(5.545)	(3.213)	(2.993)	(2.574)
Mb	0.001	0.001	0.001	0.001
	(0.503)	(0.415)	(0.531)	(0.529)
Lev	-0.316***	-0.199***	-0.207***	-0.099**
	(-11.945)	(-4.049)	(-8.975)	(-2.255)
Ppe	-0.042	-0.097	0.026	0.001
	(-1.020)	(-1.251)	(0.720)	(0.023)
Invent	0.010	0.070	-0.058	-0.021
	(0.203)	(0.626)	(-1.421)	(-0.218)
Intang	-0.117	0.261	0.008	-0.023
	(-1.232)	(1.146)	(0.092)	(-0.120)
Age	-0.146	-0.105	-0.162	0.094
	(-1.105)	(-0.616)	(-1.416)	(0.584)
Soe	-0.025	-0.005	-0.015	0.011
	(-1.209)	(-0.144)	(-0.845)	(0.452)
shrcr1	0.115*	0.079	0.108*	0.001
	(1.794)	(0.709)	(1.926)	(0.013)
Boardsize	0.023	-0.012	0.016	0.036
	(0.738)	(-0.230)	(0.597)	(0.769)
Iboard	0.073	-0.079	-0.031	0.061
	(0.712)	(-0.466)	(-0.349)	(0.421)
Firm	yes	yes	yes	yes
Ind	yes	yes	yes	yes
Year	yes	yes	yes	Yes
Constant	-0.811*	-0.415	-0.061	-0.467
	(-1.870)	(-0.649)	(-0.161)	(-0.758)
Observations	1,247	1,242	1,247	1,242
Adj R2	0.535	0.315	0.485	0.339
p-value	0.088		0.090	

## Conclusion

Under the background of the declining economic growth and reducing tax revenue in China, it is of great significance to combat corporate tax avoidance behavior and to ensure the government’s effective operation. In the past, the literature mainly focused on the impact of the government’s financial behavior and corporate governance structure on corporate tax avoidance but ignored the effect of securities supervision on tax avoidance. In this paper, we examine the impact of the tax-related comment letters on the tax avoidance behavior of listed companies from 2014 to 2019. The results show that firms that engage more in tax avoidance are more likely to receive tax-related comment letters, and the tax-related comment letters can curb firms’ tax avoidance behavior in subsequent years. Further research finds that the number of questions, the frequency of inquiries, and the type of reply letters play a role in reducing tax behavior. After considering the influence of government behavior, we discover that the effect of tax-related comment letters on tax avoidance management is more significant for state-owned and non-state-owned enterprises that have connections with political authority. There is a substitution relationship between tax collection and tax-related comment letters on tax avoidance monitoring.

The conclusion of this paper has the following implications: (1) The securities supervision could potentially strengthen the effect of reducing corporate tax avoidance. Although the Chinese government has been committed to improving the tax collection and administration, the collection management resources and tax capacity are always limited. The conclusion of this paper shows that securities supervision also has the function of inhibiting corporate tax avoidance. It is a beneficial supplement to the sharing of the tax authorities’ collection pressure and ensuring the government’s financial resources. (2) The government intervention in corporate behavior should be further reduced. This paper shows that the governmental “implicit guarantee” and political connection reduce the tax avoidance monitoring effect of the comment letters, and to some extent hinder the effective play of the securities regulatory supervision. Therefore, it is necessary to deepen the reform of the relationship between the government and the capital market, clarify the boundary between the two, reduce the government intervention in the market and better play the governmental regulatory function.

## Supporting information

S1 File Original data source for this paper. (DTA)

S1 Script Runnable STATA source code to replicate the results by using plos_data.dta data. (DO)
